# Complications and revisions in anatomic and reverse short stem shoulder arthroplasty

**DOI:** 10.1007/s00402-023-04802-4

**Published:** 2023-02-16

**Authors:** Markus Loew, Marc Schnetzke, Sophia Kappes, Thomas Bruckner, Anna-Katharina Nolte

**Affiliations:** 1 German Joint Centre, ATOS Clinic Heidelberg, Bismarckstraße 9, 69115 Heidelberg, Germany; 2 Institution for Medical Biometrics, Heidelberg University Hospital, INF 130.3, 69120 Heidelberg, Germany; 3 Department of Orthopaedics, Heidelberg University Hospital, Schlierbacher Landstraße 200a, 69118 Heidelberg, Germany

**Keywords:** Anatomic total shoulder arthroplasty, Reverse total shoulder arthroplasty, Short stem, Risk factors

## Abstract

**Introduction:**

One current trend in the field of shoulder arthroplasty is a design shift to shorter and metaphyseal fixed humeral stem components. The aim of this investigation is to analyze complications resulting in revision surgery after anatomic (ASA) and reverse (RSA) short stem arthroplasty. We hypothesize that complications are influenced by the type of prosthesis and indication for arthroplasty.

**Materials and methods:**

A total of 279 short stem shoulder prostheses were implanted by the same surgeon (162 ASA; 117 RSA), and 223 of these prostheses were implanted as primary procedures; in 54 cases, arthroplasty was performed secondary to prior open surgery. Main indications were osteoarthritis (OA) (*n* = 134), cuff tear arthropathy (CTA) (*n* = 74) and posttraumatic deformities (PTr) (*n* = 59). Patients were evaluated at 6 weeks (follow-up 1; FU1), 2 years (FU2) and the time span of the last follow-up defined as FU3 with a minimum FU of 2 years. Complications were categorized into early complications (within FU1), intermediate complications (within FU2) and late complications (> 2 years; FU3).

**Results:**

In total, 268 prostheses (96.1%) were available for FU1; 267 prostheses (95.7%) were available for FU2 and 218 prostheses (77.8%) were available for FU3. The average time for FU3 was 53.0 months (range 24–95). A complication leading to revision occurred in 21 prostheses (7.8%), 6 (3.7%) in the ASA group and 15 (12.7%) in the RSA group (*p *< 0.005). The most frequent cause for revision was infection (*n* = 9; 42.9%). After primary implantation, 3 complications (2.2%) occurred in the ASA and 10 complications (11.0%) in the RSA group (*p* < 0.005). The complication rate was 2.2% in patients with OA, 13.5% in CTA and 11.9% in PTr.

**Conclusions:**

Primary reverse shoulder arthroplasty had a significantly higher rate of complications and revisions than primary and secondary anatomic shoulder arthroplasty, respectively. Therefore, indications for reverse shoulder arthroplasty should be critically questioned in each individual case.

## Introduction

Anatomic (ASA) and reverse (RSA) shoulder arthroplasty has become well-established and safe surgical procedures in the treatment of degenerative diseases of the glenohumeral joint [39]. One current trend in the field of shoulder arthroplasty is a design shift from cemented standard stem prostheses to cementless, shorter and metaphyseal fixed humeral stem components [20, 28]. Advantages of the new design are seen in the bone stock preserving implantation technique, especially in the event of revision [13, 21]. So far, clinical short- to midterm results for both short stem TSA and RSA are promising [11, 32, 34, 36, 44]. As a matter of concern, however, some authors reported the risk of stress shielding due to bone adaptions [29, 33, 35, 36, 38] and stem subsidence [43], which might facilitate stem loosening in the future.

Another trend is a more frequent use of RSA for primary joint replacement within the past 10 years. In the 2020 annual report of the Australian Orthopaedic Association National Joint Replacement Registry [1], one of the largest databases for shoulder arthroplasty worldwide, the proportion of RSA has increased from 42% in 2009 to 80.4% in 2019. In the latest report, which contains data up to December 2019, 44,561 total shoulder replacements were considered of which 32% were ASA and 68% were RSA, respectively. According to the German Shoulder Arthroplasty Registry (DVSE) report of 2020, comparable numbers can be found with 24% ASA and 76% RSA in 2020[20]. This new trend toward more frequent implantation of RSA was recently explained by a rising proportion of active elderly patients electing for RSA [20]. Another reason might be an expansion of indications for RSA from historical indications like cuff tear arthropathy (CTA) in elderly patients [42] and revision cases [15] to shoulders with B2 glenoid types according to the classification of Walch et al. [2, 8, 9, 18, 45, 46] and arthritic shoulders with an intact rotator cuff [47, 48].

The aim of this investigation is to record and analyze complications resulting in revision surgery after anatomic (ASA) and reverse (RSA) short stem arthroplasty. We hypothesize that complications are influenced by the type of prosthesis (ASA or RSA) and indication for joint replacement.

## Methods

A retrospective comparative study was conducted on prospectively collected data of 162 short stem ASA and 117 short stem RSA performed at a single specialized shoulder center between January 2013 and December 2019. Institutional review board approval was obtained prior to the start of the study.

All surgeries were performed by the senior author (blinded for review). Inclusion criteria were osteoarthritis, OA; cuff tear arthropathy, CTA; posttraumatic deformities, PTr, rheumatoid arthritis, RA and avascular humeral head necrosis, AVN. Patients with revision surgery after prior arthroplasty were excluded from this study.

A cementless short stem system (AscendTM Flex, Wright Medical, Memphis, TN, USA) was used in all cases.

The implantation of ASA or RSA was defined as the index surgery. Osteoarthritis with an intact RC was the main indication for ASA in this series. In rare cases, elderly patients with OA and debatable compliance for complex rehabilitation and/or muscle atrophy were treated with RSA despite an intact rotator cuff. Glenoid deformity, according to Walch’s classification system [45], was not a criterium for RSA in any case. The distribution of glenoid types among 122 patients operated for primary OA with an intact rotator cuff was: Type A1: 3.3%, Type A2: 31.1%, Type B1: 48.4%, Type B2: 14.0%, Type A3: 0.8%, Type C: 2.5%.

All patients with cuff tear arthropathy were treated with RSA. In shoulders with posttraumatic sequelae (PTr), the decision for ASA or RSA was made depending on the type of deformities and the situation of the rotator cuff.

All procedures were further categorized into primary or secondary indications. Primary indications (PI) were defined as joint replacements without previous major operations. Patients with prior minor surgical procedures, such as arthroscopic decompression, rotator cuff or Bankart repair, were also classified as PI. Arthroplasties following prior major open procedures, such as bone reconstructive interventions, open stabilization procedures and open rotator cuff repair, were classified as secondary indications (SI).

All patients were followed up at six weeks (FU1) and two years (FU2) after the index surgery. Regular clinical controls (every 2 years) were recommended by the surgeon for each patient. Controls with a minimum FU of more than 2 years were defined as FU3. All patients who had missed their last regular checks were invited for clinical examination. Patients, who were not able to travel due to the COVID-19 pandemic, unacceptable distance to the hospital or due to their medical condition were called by phone and asked for complications or revisions by one of the authors (blinded for review).

All complications that were followed by revision surgery were analyzed. Complications were categorized into early complications (within the first 6 weeks; FU1), intermediate complications (within the first 2 years; FU2) and late complications (after more than two years; FU3). Cases of revision due to postoperative infections were categorized as early-onset (< 3 months)[23] and late-onset (> 3 months) infections [24]. The impact of type of prosthesis (anatomic or reverse) and indication (primary or secondary) on revision rates was analyzed. The Fisher–Boschloo test [25] was used for statistical evaluation. The Fisher–Boschloo’s test is a statistical hypothesis test for analyzing 2 × 2 contingency tables. It examines the association of two Bernoulli distributed random variables and is a uniformly more powerful alternative to Fisher's exact test.

### Surgical technique and implant

Anatomic implant and technique: A deltopectoral approach was used in all cases. Resection of the humeral head was performed in a free-hand technique according to the individual conditions. The smallest fitting trial stem (compactor) was implanted into the metaphyseal cancellous bone and found appropriate in size when the surgeon was unable to rotate the stem with three fingers. An uncemented humeral short stem (Ascend Flex ™, Wright Medical, Memphis, TN, USA) was then implanted. A cemented keeled glenoid (Perform™ Glenoid, Wright Medical, Memphis, TN, USA) was used in all patients. In B1 or B2 glenoids, according to Walch’s classification [45], partial correction of retroversion was performed by restrained eccentric reaming. Posterior glenoid augmentation was not performed in any case.

Reverse implant and technique: The humeral stem was implanted in the same fashion as described above at 130° of inclination and 20° of retroversion. The platform was placed in the most medialized position and the optimal inlay size was individually selected. Based on the diameter of the resected humeral head, the size of the glenosphere was chosen. The glenoid implant was anchored to the bone with four screws. From 2013 until 2016, a standard glenosphere (Wright Medical, Memphis, TN, USA) was implanted and, since 2017, the Aequalis Perform Reversed Glenosphere (Wright Medical, Memphis, TN, USA) was used for all cases. The detailed surgical techniques have been described before [37].

## Results

**Fig. 1:**
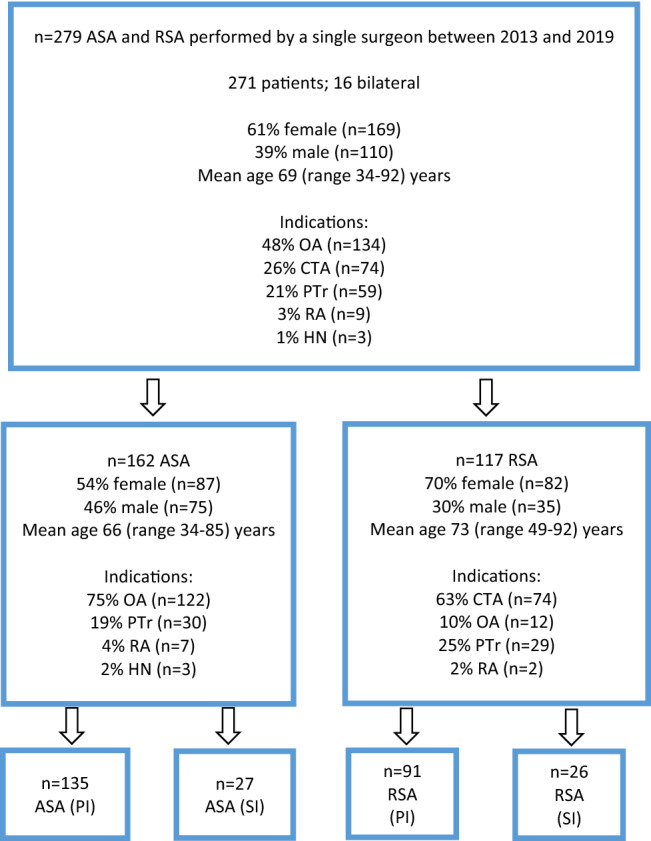
Demographics and characteristics. ASA = anatomic total shoulder arthroplasty; RSA = reverse shoulder arthroplasty; OA = osteoarthritis; PTr = post-traumatic; RA = rheumatoid arthritis; AVN = avascular humeral head necrosis; CTA = cuff tear arthritis; PI = primary indication; SI = secondary indication

The overall cohort included in this study consisted of 279 prostheses: 162 ASA and 117 RSA (271 patients; 16 bilateral procedures). A total of 169 prostheses were used in females (61%), and 110 in males (39%). Mean age was 69 (range 34–92) years. The right shoulder joint was replaced in 186 cases (67%) and the left in 93 (33%) cases. The most common indication for arthroplasty was OA (*n* = 134; 48.0%) followed by CTA (*n* = 74; 26.4%) and PTr (*n* = 59; 21.1%). Arthroplasty secondary to major open procedures (secondary indication; SI) was performed in 54 cases (19.3%). OA was the main indication for primary ASA (88.9%) and CTA for primary RSA (81.3%). In 12 cases (10%), RSA was used for primary OA with an intact rotator cuff. Demographics and characteristics are demonstrated in Fig. 1.

A total of 268 prostheses (96.1%) were available for FU1 (ASA 97.5%; RSA 94.0%) and 267 prostheses (95.7%) for FU2 (ASA 96.9%; RSA 94.0%); 217 prostheses (77.8%) had a minimum FU of two years (ASA 82.7%; RSA 71.0%) with a mean of 53.0 (range 24–95) months; 42 patients (15%) were unavailable via phone contact and 11 patients (3.9%) had died in the meantime.

### Complications

The overall revision rate for the entire observation period was 3.7% (6 cases) in ASA and 12.7% (15 cases) in RSA. Infection was the most often reason for revision surgery (9 cases; 42.9%): late-onset infection occurred in six cases (29%) and early-onset infections occurred in three cases (14%). Proven bacteria were: *Staphilococcus epidermidis (two cases)*, *Staphilococcus capitis (one case), cutibacterium acnes (one case)* and *Pseudomonas aeruginosa (one case)*. The second most frequent complication was aseptic component loosening in three cases (14.3%), exclusively occurring in the RSA group. Detailed indications, complications and revisions are demonstrated in Tables 1 and 2.

**Table 1:** Complications after ASA (anatomic total shoulder arthroplasty)

Index diagnosis	Primary (PI) or secondary indication (SI)	Detail of complications	Time from index surgery until first complication	Detail of revision
PTr	SI	LOI	7 months	2 step revision to RSA
PTr	SI	LOI	13 months	2 step revision to RSA
PTr	SI	Secondary cuff deficiency	15 months	Revision to RSA
OA	PI	Periprosthetic fracture	19 months	ORIF
OA	PI	SSC deficiency	25 months	Revision to RSA
OA	PI	LOI	52 months	2 step revision to RSA

*PTr*  post-traumatic, *OA*  osteo-arthritis, *PI* primary indication, *SI*  secondary indication, *ORIF*  open reduction and internal fixation, *LOI*  late-onset infection

**Table 2:** Complications after RSA (reverse shoulder arthroplasty)

Index diagnosis	Primary (PI) or secondary indication (SI)	Detail of complications	Time from index surgery until first complication	Detail of revision
CTA	PI	Hematoma	5 days	Debridement
PTr	SI	Hematoma	22 days	Debridement
CTA	SI	EOI	1 month	Unknown (external center)
CTA	SI	LOI	2 months	Unknown (external center)
CTA	PI	Glenosphere dislocation	2 months	Replacement glenosphere, bone graft
CTA	PI	EOI	3 months	Unknown (external center)
CTA	PI	LOI	5 months	2 step revision to RSA
PTr	PI	LOI	7 months	2 step revision to RSA
PTr	SI	Periprosthetic fracture	13 months	Unknown (external center)
PTr	SI	Acromion stress fracture	16 months	ORIF
CTA	PI	Stem loosening	18 months	Unknown (external center)
CTA	PI	Glenosphere dislocation	18 months	Replacement glenosphere, bone graft
CTA	PI	Glenosphere loosening	23 months	Replacement glenosphere, bone graft
RA	PI	Both component loosening	40 months	Hemiarthroplasty
CTA	PI	LOI	60 months	2 step revision to RSA

*CTA*  cuff tear arthropathy, *PTr*  post-traumatic, *RA*  rheumatoid arthritis, *PI*  primary indication, *SI*  secondary indication, *ORIF*  open reduction and internal fixation, *LOI*  late-onset infection, *EOI*  early-onset infection

Among 122 patients treated with an ASA for OA with an intact rotator cuff, revision was performed in just one case due to secondary rotator cuff deficiency.

No complications occurred among patients who received RSA for OA with an intact rotator cuff.

### Early complications (< 6 weeks FU)

A total of 268 prostheses (96.1%) were available for FU1. Early complications occurred in three cases (1.1%), exclusively in the RSA group (Table 2).

### Intermediate complications (6 weeks – 2 years FU)

A total of 267 prostheses (95.7%) were available for FU2. Intermediate complications occurred in 14 cases (5.2%). Four complications were found after ASA (2.5%) and 10 complications (9%) after RSA (Tables 1 and 2).

### Late complications (> 2 years FU)

A total of 217 prostheses (77.8%) were available for FU3. Late complications occurred in four cases (1.8%). Two complications (1.5%) were found after ASA, and two complications (2.4%) were found after RSA (Tables 1 and 2).

### Complications and revisions depending on indications for index surgery

OA was the indication for arthroplasty in 134 cases; 122 patients (91.1%) received ASA, and 12 patients (9%) received RSA. Three complications (2.5%) in the ASA group required revision surgery: a periprosthetic fracture, a secondary rotator cuff failure (M. subscapularis defect) and a late-onset infection.

In 74 cases, arthroplasty was performed for CTA and all these cases were treated with RSA. Ten complications (13.5%) required revision surgery: postoperative hematoma (*n* = 1), traumatic component dislocation (*n* = 1), early-onset infection (*n* = 2), late-onset infection (*n* = 3), stem loosening (*n* = 1), atraumatic glenosphere dissociation (*n* = 1) and glenosphere loosening (*n* = 1).

In 59 cases, arthroplasty was performed for PTr. 30 patients received ASA (50.8%) and 29 patients received RSA (49.2%). Three complications occurred in the ASA group: late-onset infection (*n* = 2) and secondary rotator cuff deficiency due to non-healing of the subscapularis tendon (*n* = 1). Four complications occurred in the RSA group: late infection (*n* = 1), periprosthetic fracture (*n* = 1), hematoma (*n *= 1) and acromion stress fracture (*n* = 1).

A total of 226 patients (80.7%) had primary indications (PI). Of those, 135 received ASA (59.7%) and 91 received RSA (40.3%). Three complications in the ASA group and 10 complications in the RSA group led to revision surgery.

A total of 53 patients (19.0%) had secondary indications (SI). Of those, 27 received ASA (51.0%) and 26 received RSA (49.0%). Three complications in the ASA and 5 complications in the RSA group led to revision surgery. In nine cases, arthroplasty was performed for patients with rheumatoid arthritis. ASA was used in seven (78%) and RSA in two cases (22%). One complication in the RSA group led to revision surgery. Three patients (1%) were indicated for ASA due to humeral head necrosis. After AVN, no complications were recorded.

### Statistical analysis

Using the Fisher–Boschloo test, a significant difference was found between ASA and RSA. A complication requiring revision occurred in 6 ASA (3.7%) and in 15 RSA (12.7%) (*p* < 0.005). After primary implantation, three complications (2.2%) occurred in the ASA and 10 complications (11.0%) in the RSA group (*p* = 0.005). The difference between ASA and RSA could not be confirmed for secondary indications (SI). After SI, three complications (11.1%) occurred in the ASA and five complications (18.5%) in the RSA group (*p* = 0.649). The complication rate was 2.5% after OA, 13.5% after CTA, 11.9% after PTr and 14.8% after SI. Mean time until revision was 50.7 months for ASA and 53.4 months for RSA.

## Discussion

A current trend in the field of shoulder arthroplasty is a design shift from cemented standard stem prostheses (length > 100 mm) to uncemented, metaphyseal fixed short humeral stem components [20, 28] with potential advantages in the event of revision due to a bone-preserving implantation technique [13, 21]. Promising clinical results for both short stem ASA and short stem RSA have been reported; however, only short- to medium-term data exist as short stem arthroplasty has only become popular recently [11, 32].

This study aimed to record and analyze complications resulting in revision surgery among 162 short stem ASA and 117 short stem RSA. Since the senior author performed all arthroplasties in this study, complications due to the bias by surgeons with different levels of experience, as previously shown [3], are most likely to be excluded.

The results demonstrate a revision rate of 3.7% (6 cases) after short stem ASA. These results are in line with a revision rate of 4.0% after short stem ASA reported by Schnetzke et al.[34] in a systematic review among ten included studies with an average follow-up of 20–64 months. Compared to reported revision rates after standard stem ASA, the complication rate after short stems is relatively low. Deshmukh et al.[10] described a revision rate of 22.0% after standard stem ASA at a minimum follow-up of 10 years and Gonzales et al. [16] reported a revision rate of 17.3% after ASA in their systematical review. The results of the current study imply a rather low revision rate after short stem arthroplasty, but due to a lower observation period, higher revision rates must be expected with longer follow-up.

Furthermore, the study found a revision rate of 12.7% (15 cases) after short stem RSA. Compared to a 4.9% revision rate after short stem RSA in a systematic review with an average follow-up of 20–99.6 months among 10 included studies, the revision rate in this study is somewhat higher [44]. Also, the reported revision rate in this study appears to be higher than revision rates after standard stem RSA [5, 14, 17, 22, 41, 50]. As an example, Boileau et al. [5] reported a revision rate of 4.0% after the implantation of 143 Grammont-style BIO (bio-increased offset) RSA at a mean observation time of 75 months and Sirveaux et al.[41] found a revision rate of 3.75% at a mean FU of 44 months in their multicenter study using the same type of prosthesis.

An explanation for higher revision rates among the present study cohort might be the inclusion of patients with posttraumatic sequelae and major open surgery before the index surgery, which made the cohort more heterogeneous than cohorts in other studies that predominantly included cuff tear arthropathy [26]. A study by Ascione et al. included various diagnoses and a comparable revision rate of 10.0% was found after the implantation of the same type of short stem RSA in 100 cases with a mean follow-up of 32.6 months. Also, the present analysis revealed that the mean time until revision was 53.4 months after the implantation of RSA, which is more than the maximum FU of the most published studies on short stem RSA [44].

In the past, high rates of bone adaptions [29, 33, 35, 36, 38] and stem subsidence [43, 49] have called into question the long-term stability of short stem prostheses. In the present study, stress shielding was not analyzed in detail, but no case of aseptic loosening of the humeral component was seen after ASA, and only two cases of stem loosening (1.7%) were found after RSA.

The present study is one of the first to compare revision rates of short stem ASA and short stem RSA. The analysis identified reverse shoulder arthroplasty as one risk factor for higher complication rates.

A complication requiring revision occurred significantly more often after short stem RSA compared to short stem ASA. Also, the revision rate after primary implantation of RSA was significantly higher compared to primary implantation of ASA. Among the study cohort, 54 patients were treated with an arthroplasty secondary to complex open procedures, such as bone reconstructive interventions, open stabilization procedures and open rotator cuff surgery.

Due to the heterogeneity in this group, a statistical comparison must be drawn with caution. However, the difference between ASA and RSA could not be confirmed for these secondary indications. Other studies in the past have shown comparable complication and revision rates for standard stem ASA and RSA [12, 19, 47].

As the results of this study imply higher revision rates after RSA, the increasing usage of RSA, as demonstrated in national and international shoulder arthroplasty registries [1, 20], should be critically discussed. Kircher et al. [20] explained the rising numbers of RSA with an increasing proportion of active elderly patients electing for RSA due to CTA, proximal humeral fractures and irreparable rotator cuff lesions.

Historically, the idea of RSA was to restore mobility and function in shoulders with cuff tear arthropathy [4, 42]. In this situation, the deltoid muscle is able to replace the rotator cuff to a large extent [6]. Nowadays, however, an increasing number of surgeons and authors consider RSA beneficial over ASA based on age of the patient and the likelihood of developing a rotator cuff deficiency in the future [47, 48]. Some authors considered secondary rotator cuff degeneration as the most common complication after ASA leading to glenoid loosening through the “rocking horse” mechanism [7, 47, 48]. Young et al. [48] reported a secondary rotator cuff dysfunction rate of 16.8% nine years after ASA. In their study, secondary rotator cuff dysfunction was associated with worse clinical outcomes; however, revision rates among the study cohort were not significantly different for patients with or without an intact rotator cuff.

On the other hand, Raval et al. [30] demonstrated that the preoperative presence of a partial rotator cuff tear was not associated with worse clinical outcomes 5.8 years after ASA. Moreover, the authors found a survival rate of over 90% at five years, which is comparable to other survival rates of ASA in the literature [40].

In the present study, secondary rotator cuff deficiency led to revision surgery in only one case, 25 months after ASA for OA with an intact rotator cuff. Another patient received ASA in a posttraumatic situation and was revised for rotator cuff deficiency due to non-healing of the subscapularis tendon 15 months after refixation.

Although evaluation of the rotator cuff before arthroplasty is crucial and especially fatty infiltration of the infraspinatus was shown to be predictive for a secondary rotator cuff failure [48], the authors of this article think that the implantation of RSA must be critically questioned when the rotator cuff is not torn by the time of arthroplasty. It should be reserved for patients with cuff tear arthropathy or osteoarthritis with massive rotator cuff tears.

Furthermore, controversy exists as to which type of arthroplasty is more useful in primary arthritis with an intact rotator cuff but biconcave glenoid deformities.

Walch et al. [46] reported a revision rate of 16.3% among 92 ASA implanted in shoulders with B1 and B2 glenoids at an average follow-up of 77 months, where revision was due to glenoid loosening, posterior instability or soft tissue problems. The authors thus recommended RSA as the preferred treatment option for biconcave glenoids [46].

Since then, in cases with biconcave glenoids in primary arthritis, the concept of RSA has competed with that of ASA with adjusted correction of the position of the glenoid component. In a recently published systematic review, Reahl et al. [31] compared the midterm clinical outcomes of ASA and RSA for B2 glenoids with an intact rotator cuff. Both groups showed improvement in patient-reported outcome scores and pooled complication rates (9% after ASA and 6% after RSA) as well as revision rates (2% after ASA and 1% after RSA). Single reports about good functional outcomes and low rates of glenoid component loosening after the implantation of RSA in shoulders with B2 glenoids and an intact rotator cuff exist [27]. However, randomized controlled trials are crucial to demonstrate the long-term superiority of RSA over ASA in these special cases.

As far as we know, no controlled comparative study has been performed about this issue and there is still no proof of lower complication and revision rates after RSA for arthritis with biconcave glenoids. The superiority of one of the two implants is therefore not demonstrated.

In fact, this study contributes nothing new to this question. It does, however, show a very low complication rate in cases with biconcave glenoids after restrained correction of the retroversion of the glenoid. On the other hand, the few cases in which an RSA was implanted in osteoarthritis with an intact rotator cuff did not show a higher complication rate.

However, it should be borne in mind that in the case of a glenoid failure, conversion from ASA to RSA is an easier retreat option than revision in a loosened glenosphere.

### Limitations

The results of the two types of prostheses, ASA and RSA, used in this study are clearly not comparable. On the one hand, it is a retrospective analysis and, on the other hand, heterogeneous collectives and indications obviously lead to an indication bias. A multicenter prospective randomized study would have to be performed to clearly demonstrate the superiority of one of the systems.

## Conclusion

Revision rates in short stem arthroplasty are generally low. Primary reverse shoulder arthroplasty was identified as a risk factor for complications requiring revision surgery. Therefore, indications for reverse shoulder arthroplasty should be critically questioned in each individual case.

## Data Availability

The datasets generated during and/or analysed during the current study are available from the corresponding author on reasonable request.
